# Prevalence of Bovine Tuberculosis in India: A systematic review and meta‐analysis

**DOI:** 10.1111/tbed.12915

**Published:** 2018-06-08

**Authors:** Sreenidhi Srinivasan, Laurel Easterling, Bipin Rimal, Xiaoyue Maggie Niu, Andrew J. K. Conlan, Patrick Dudas, Vivek Kapur

**Affiliations:** ^1^ Department of Animal Science The Pennsylvania State University University Park Pennsylvania USA; ^2^ The Huck Institutes of the Life Sciences The Pennsylvania State University University Park Pennsylvania USA; ^3^ Department of Statistics Eberly College of Science The Pennsylvania State University University Park Pennsylvania USA; ^4^ Disease Dynamics Unit Department of Veterinary Medicine University of Cambridge Cambridge UK

**Keywords:** bovine tuberculosis, buffaloes, cattle, control program, cows, India, meta‐analysis, prevalence, review

## Abstract

Bovine tuberculosis (bTB) is a chronic disease of cattle that impacts productivity and represents a major public health threat. Despite the considerable economic costs and zoonotic risk consequences associated with the disease, accurate estimates of bTB prevalence are lacking in many countries, including India, where national control programmes are not yet implemented and the disease is considered endemic. To address this critical knowledge gap, we performed a systematic review of the literature and a meta‐analysis to estimate bTB prevalence in cattle in India and provide a foundation for the future formulation of rational disease control strategies and the accurate assessment of economic and health impact risks. The literature search was performed in accordance with PRISMA guidelines and identified 285 cross‐sectional studies on bTB in cattle in India across four electronic databases and handpicked publications. Of these, 44 articles were included, contributing a total of 82,419 cows and buffaloes across 18 states and one union territory in India. Based on a random‐effects (RE) meta‐regression model, the analysis revealed a pooled prevalence estimate of 7.3% (95% CI: 5.6, 9.5), indicating that there may be an estimated 21.8 million (95% CI: 16.6, 28.4) infected cattle in India—a population greater than the total number of dairy cows in the United States. The analyses further suggest that production system, species, breed, study location, diagnostic technique, sample size and study period are likely moderators of bTB prevalence in India and need to be considered when developing future disease surveillance and control programmes. Taken together with the projected increase in intensification of dairy production and the subsequent increase in the likelihood of zoonotic transmission, the results of our study suggest that attempts to eliminate tuberculosis from humans will require simultaneous consideration of bTB control in cattle population in countries such as India.

## 1 INTRODUCTION

Bovine tuberculosis (bTB) is a chronic granulomatous inflammatory disease that is predominantly caused by *Mycobacterium bovis*. While primarily affecting bovines, the pathogen has a broad host range that includes humans. It has been estimated that *M. bovis* causes ~10% of the total human TB cases in developing countries and subsequently poses a significant threat to global health (Olea‐Popelka et al., 2014) (Etchechoury et al., 2010) (“OIE, Bovine Tuberculosis: General Disease Information sheets,”). Prior to mandatory pasteurization of milk in many countries, *M. bovis* accounted for ~25% of all TB cases in children (Roswurm & Ranney, 1973). In addition to being a threat to public health, bTB is also a major economic concern, costing an estimated USD 3 billion worldwide annually due to losses from reduced cattle productivity, culling and movement and trade restrictions (Waters, Palmer, Buddle, & Vordermeier, 2012).

Bovine TB is well controlled in most developed countries where national control programmes have been implemented, although complete eradication and maintenance of bTB‐free status are challenging given the potential of spillover from wildlife reservoir hosts. Such control programmes for bTB were successfully adopted over a century ago in many developed countries by applying test and cull strategies, resulting in enormous benefits to human health and more than 10‐fold return on investment in animal productivity (Olmstead & Rhode, 2004). In contrast, bTB remains endemic in developing countries like India that lack disease control programmes because of the associated economic costs and social barriers to test and cull strategies. This current level of endemicity is likely to increase in the coming years due to a confluence of factors including the growing intensification of dairy and cattle farming and increased emphasis on improving animal productivity in these countries.

In conjunction with possessing the largest population of cattle in the world (nearly 300 million cows and buffaloes) (Basic Animal Husbandry and Fisheries Statistics, Government of India 2017), India's lack of a control programme poses a potential threat for bTB infection and transmission worldwide. In the absence of a national surveillance programme, accurate prevalence data are lacking and, to our knowledge, there has thus far not been a comprehensive review of the existing literature to determine an estimate of the overall prevalence of bTB in the country. Such an estimate will prove crucial in future efforts to accurately assess risk and inform policy for the development of effective control strategies. In this systematic review and meta‐analysis, we sought to address this critical gap and determine the overall prevalence of bTB in the cattle of India. This systematic review conforms to Preferred Reporting Items for Systematic Reviews and Meta‐analyses (PRISMA) guidelines (Liberati et al., 2009).

## 2 METHODS

### 2.1 Literature search strategy

A systematic search for published articles reporting prevalence data for bTB in cows and buffaloes in India was conducted on 11th September 2017. The four databases used in our search (CAB Direct, Web of Science, Web of Science Biological Abstracts and PubMed) were selected in order to comprehensively capture articles published in both international and local journals and minimize journal biases. After examining common MeSH terms for pre‐identified and relevant publications, the following search terms were used across all four databases: ((“mycobacterium bovis” OR tuberculosis) AND (cows OR cattle OR bovine) AND (epidemiolog* OR prevalen* OR inciden* OR surve*) AND (India)). No restrictions were placed on the date of publication. The citation software program EndNote X8 (Clarivate Analytics, Philadelphia, PA) was used to organize and remove duplicate articles between the databases. Additional articles were also identified manually from the reference lists of articles generated in the database search.

### 2.2 Study inclusion criteria

The inclusion/exclusion criteria for data extraction are detailed in Table 1. Included studies reported the prevalence of bTB in cows and/or buffaloes in India based on commonly accepted methods for the diagnosis of bTB. More specifically, studies whose main objectives were not to determine bTB prevalence but required a preliminary prevalence study for determining initial disease status were included as long as data were reported and animals were not pre‐selected for bTB symptoms. Prevalence studies that examined the effects of bTB control strategies were excluded in order to avoid the introduction of potential sampling bias, as the primary aims of these studies were to compare the effectiveness of control strategies. For instance, Dhanda et al. have reported an increase in prevalence in herds at Puri, Orissa, from 9.1% in 1937 to 84.7% in 1942 (Dhanda & Lall, 1959). The cattle populations that were tested were part of farms that did not practice any bTB control strategies. We believe that inclusion of studies conducted on pre‐selected herds as opposed to randomly sampled prevalence studies would not be truly representative of the prevailing prevalence in the region. Also, all other publications that did not precisely fit the main exclusion categories were excluded within the “Other” category. Finally, all included studies were cross‐sectional in nature.

**Table 1:** Study inclusion/exclusion criteria

Inclusion	Exclusion
Cross‐sectional prevalence study	Wrong type of study: not a cross‐sectional study or animals chosen for bTB symptoms
Study conducted in India	Study conducted elsewhere
Tested for *Mycobacterium bovis* using standard diagnostic tests	Study not addressing bTB
Any breed of cow or buffalo	Study neither performed on cow nor on buffalo
Reported the prevalence of bTB and the number of total animals screened	No statistics reported
In English	Language limitation: Not in English
Full text of publication obtained	Full text unavailable
	Other

*Note*. Criteria for study inclusion or exclusion to our systematic review on the prevalence of bTB in India.

### 2.3 Data extraction

Before beginning data extraction, a template was created based on population demographics and other conditions common to bTB prevalence studies. The data set recording general study characteristics included author, publication year, study period, location of study, diagnostic test used, criteria for positivity, sample size, prevalence by different production system, overall prevalence for cow and buffalo combined, overall prevalence for specific cattle breeds, and overall prevalence for male and female animals. Headings for prevalence data broken down by more specific characteristics were production system (organized farm, rural, Gaushala and other), cow breed (exotic, indigenous and cross‐bred), sex, age (younger or older than 6 months) and species (cow versus buffalo). Data extracted from studies’ individual farm‐level data by each of the three of the authors (SS, LE and BR) were assigned to different strata targeted in this study. The determination of bTB infection status was accepted as reported by the studies.

A pilot test on 20 randomly selected papers was performed in order to test the inclusion and exclusion criteria and finalize the data extraction form. For the formal review of all articles generated, an initial screening for inclusion was made based on the titles and abstracts, and publications that were clearly based on different species, countries or diseases were immediately excluded. Otherwise, full texts were read for any prevalence data that could be extracted. Three of the authors (SS, LE and BR) independently reviewed all publications before comparing their respective data forms. When discrepancies were found amongst the forms, the authors (SS, LE and BR) collectively discussed their reasoning before reaching a final consensus. All studies included and excluded are publicly available at https://doi.org/10.18113/d37s9x.

### 2.4 Statistical analysis

All quantitative analyses were performed in RStudio (version 1.0.143) (“R core team, R: A language and environment for statistical computing.,” R core team 2016) where the “meta” package was used to estimate models (Schwarzer, 2007) (Viechtbauer, 2010). Codes used for the statistical analyses are publicly available at https://doi.org/10.18113/d37s9x. The prevalence estimates from individual studies were logit‐transformed, and the pooled prevalence was estimated using meta‐analytic models. Cochran's Q statistic (Cochran, 1954) was computed to test for heterogeneity, and Higgin's statistic (Higgins, Thompson, Deeks, & Altman, 2003) (*I* ^*2*^ > 50% represents at least moderate heterogeneity) helped describe the variability in the pooled prevalence estimate due to heterogeneity between studies.

A univariate screen was used to select a parsimonious set of moderator factors for multivariate analysis. Diagnostic test type was excluded from this selection procedure and forced into the final model in order to adjust for the well‐known variability in sensitivity and specificity of diagnostic tests for bTB (Farnham, Norby, Goldsmith, & Wells, 2012). Univariable meta‐regression models were estimated using both the random‐effects (RE) and fixed‐effects (FE) models for each potential moderator variable. Analysis of variance (ANOVA) tests were run on all moderators to assess their significance when compared to the full model with all other variables included. For the purposes of variable selection, given the low power of these tests and precedence set in other systematic reviews and meta‐analyses, variables with a *p*‐value < 0.25 were retained for inclusion in the final model (Sibhat et al., 2017) (Asmare et al., 2016) (Dohoo, Martin, & Stryhn, 2009).

The fit of the resulting multivariable meta‐regression models and evidence of publication bias was assessed through funnel plots, Egger's asymmetry test (Egger, Davey Smith, Schneider, & Minder, 1997) and Begg's rank correlation (Begg & Mazumdar, 1944) test.

To visualize the prevalence of bTB in the different states of India, we generated a map utilizing an open‐source library called D3.js (Data‐Drive Documents) (Bostock, Ogievetsky, & Heer, 2011). This allowed us to plot data positions via the centroids of given shapefile locations represented in the map and control graphical elements based on their values (Cleveland & McGill, 1984). We utilized a continuous log scale for circle size to represent bTB prevalence and a power function for circle lightness to represent the confidence in the prevalence estimates of each state, ratifying values to visual variables on a linear scale (Bertin, 1983).

## 3 RESULTS

### 3.1 Characteristics of included studies

**Figure 1:**
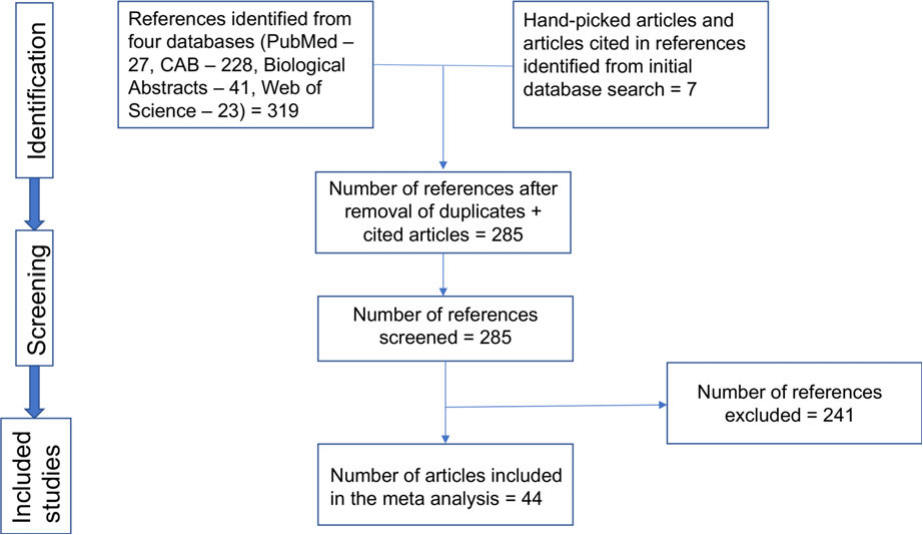
Schematic representation of the literature selection procedure for the systematic review of bTB prevalence in India [Colour figure can be viewed at http://wileyonlinelibrary.com]

From the 285 publications screened, 44 articles were included in the systematic review (Figure 1). In the instance that a publication reported prevalence data for multiple states, years, cattle breeds, species or production systems, they were considered as separate strata level data. A total of 106 strata level data were extracted from the 44 articles for meta‐analysis. For example, as seen in https://doi.org/10.18113/d37s9x, the study by Iyer (1944) has been extracted into three strata level data, the strata being the three locations in which the study was performed. The same was done for other studies that included data on different production system, breed, species, etc. These studies included in the quantitative analyses spanned from 1942 to 2016 and provided bTB prevalence data for a total sample size of 82,419 of which 29,037 were buffaloes and 53,382 were cows (Table 2).

**Table 2:** Reported bTB prevalence for included studies

Authors	Study location	Dx. Test	Sample size	Reported prevalence‡ (%)
Mallick, Aggarwal, and Dua (1942)	Punjab	DIT	1217	23.2
Iyer (1944)	Uttar Pradesh	PM Exam	250	2.4
Iyer (1944)	Maharashtra	PM Exam	120	13.3
Iyer (1944)	West Bengal	PM Exam	130	2.3
Taneja (1955)	Haryana	DIT	102	26.5
Dhanda and Lall (1959)	Gujarat	SIT	25142	16.7
Lall, Singh, and Sen Gupta (1969)	Uttarakhand	DIT	128	0.0
Lall et al. (1969)	Punjab	DIT	111	13.5
Lall et al. (1969)	Haryana	DIT	1567	2.7
Lall et al. (1969)	Bihar	DIT	169	4.7
Lall et al. (1969)	Uttar Pradesh	DIT	1418	4.9
Lall et al. (1969)	Rajasthan	DIT	727	2.6
Lall et al. (1969)	Telangana	DIT	426	1.9
Lall et al. (1969)	Maharashtra	DIT	194	1.0
Lall et al. (1969)	West Bengal	DIT	65	0.0
Lall et al. (1969)	Himachal Pradesh	DIT	177	0.6
Purohit and Mehrotra (1969)	Rajasthan	SICT	1010	1.8
Rawat and Kataria (1971)	Madhya Pradesh	DIT	1830	2.4
Nagaraja, Krishnaswamy, Adinarayanaiah, Murthy, and Nanjiah (1973)	Karnataka	DIT	3250	5.2
Joshi, Sharma, Dhillon, and Sodhi (1976)	Punjab	DIT	1081	10.5
Bali and Khanna (1979)	Haryana	SIT	663	1.4
Bali and Khanna (1979)	Haryana	SIT	624	4.6
Paily, Georgekutty, and Venugopal (1979)	Kerala	SIT	608	0.8
Appuswamy, Batish, Parkash, and Ranganathan (1980)	Haryana	Culture	308	4.6
Kulshreshtha, Jagjit, and Chandiramani (1980)	Haryana	SIT	13089	2.5
Bali and Singh (1980)	Haryana	SIT	628	2.4
Bala and Sidhu (1981)	West Bengal	NR	475	41.5
Bala and Sidhu (1981)	Haryana	NR	712	1.1
Bala and Sidhu (1981)	Uttar Pradesh	NR	732	13.1
Murti and Hazarika (1982)	Meghalaya	SICT	302	8.9
Sharma et al. (1985)	Uttar Pradesh	PM, ZN staining	1268	13.3
Bapat and Bangi (1985)	Maharashtra	SICT	2043	1.2
Maity, Deb and Pramanik (1992)	West Bengal	PM, ZN staining	1571	0.4
Sharma, Kwatra, Joshi, and Saharma (1994)	Punjab	SIT	2623	4.0
Rakesh Sisodia, Shuykla and Sisodia (1995)	Madhya Pradesh	SIT	465	9.0
Rajaram, Rao and Manickam (1996)	Tamil Nadu	SIT	1339	14.6
Mishra, Panda, and Panda (1997)	Orissa	SIT	670	3.4
Dev, Purohit, and Joshi (1998)	Rajasthan	SICT	75	10.7
Kumar, Sharma, Iyer, and Prasad (1998)	Uttar Pradesh	PM, ZN staining	1435	9.8
Aswathanarayana et al. (1998)	Karnataka	SIT	1189	25.7
Kumar and Parihar (1998)	Uttar Pradesh	PM Exam	2028	0.8
Chowdhury, Sarkar, Pal, Roy, and Chakraborty (2001)	West Bengal	PM, ZN staining	1050	3.9
Mukhopadhyay, Antony, and Pillai (2001)	Pondicherry	SICT	41	51.2
Shringi (2004)	Rajasthan	SIT	353	4.8
Singh, Gumber, Randhawa, Aradhana and Dhand (2004)	Punjab	SIT	627	9.1
Dali et al. (2004)	Maharashtra	NR**	340	6.2
Raval, Sunil, Belsare, Kanani and Patel (2006)	Gujarat	SIT	164	1.8
Raval et al. (2006)	Gujarat	SIT	167	0.0
Raval et al. (2006)	Gujarat	SIT	172	0.0
Raval et al. (2006)	Gujarat	SIT	152	3.3
Raval et al. (2006)	Gujarat	SIT	161	1.9
Ganesan (2006)	Tamil Nadu	SIT	63	65.1
Nishath and Ganesan (2006)	Tamil Nadu	SIT	63	49.2
Taggar and Bhadwal (2008)	Jammu and Kashmir	SIT	40	37.5
Phaniraja, Jayaramu, Jagadeesh and Kumar (2010)	Karnataka	SIT	2668	2.4
Aneesh, Mandeep, Katoch, Prasenjit, and Katoch (2010)	Himachal Pradesh	SIT	440	14.3
Trangadia, Rana and Srinivasan (2013)	Gujarat	SIT	2310	2.3
Trangadia et al. (2013)	Uttar Pradesh	SIT	338	0.6
Bhanu Rekha, Gunaseelan, Pawar, and Giri (2014)	Tamil Nadu	ELISA	357	4.5
Neeraja et al. (2014)	Karnataka	SIE	45	26.7
Ashish, Amit, and Deepak (2014)	Uttar Pradesh	SIT	245	14.3
Thakur, Sinha and Singh (2016)	Uttar Pradesh	SIT	442	16.1
Thakur et al. (2016)	Uttarakhand	SIT	99	0.0
Filia, Leishangthem, Mahajan, and Singh (2016)	Punjab	SICT	121	14.0

*Notes*

The reported bTB prevalence for each included study on a state‐by‐state basis. Diagnostic techniques (Dx. Tests) used were single intradermal test (SIT), single intradermal comparative tuberculin test (SICT), double intradermal test (DIT), ELISA, interferon‐gamma release assay (IGRA), multiple tests that included SIT, IGRA, and ELISA (SIE), Ziehl–Neelsen (ZN) staining and detailed post‐mortem examinations (PM). While most studies reported which Dx. test was used, some were not reported (NR) or ^‡^were unconventional. **Confidence intervals were reported for only a few studies and thus not included in the table above.

Included studies used common diagnostic techniques for bTB testing including the single intradermal test (SIT), single intradermal comparative tuberculin test (SICT), double intradermal test (DIT), enzyme‐linked immunosorbent assay (ELISA), interferon‐gamma release assay (IGRA), Ziehl–Neelsen (ZN) staining and detailed post‐mortem (PM) examinations; some studies performed multiple tests that included SIT, IGRA and ELISA (SIE). While most studies followed OIE recommended guidelines for skin test positivity at ≥4 mm after 72 hr (“International Office of Epizootics (OIE),” OIE, 2006) (the cut‐off for both SIT and SICT tests), some studies defined their cut‐off point as ≥5 mm; however, a small number of publications did not report criteria for test positivity (NR). A few studies classified animals as “doubtful” if the increase in skin thickness was between 3 and 4 mm. We did not use any cut‐off values on the number of animals for classification of the various production systems. Most included publications explicitly mentioned the type of production system that was used in their studies. In the instance that a study did not specify the production system, we did not include that study under any production system strata. To examine any effect of time on the prevalence of bTB, the study periods were separated into four time intervals: 1941–1960; 1961–1980; 1981–2000; and, 2001–2016.

### 3.2 Meta‐analysis

**Figure 2:**
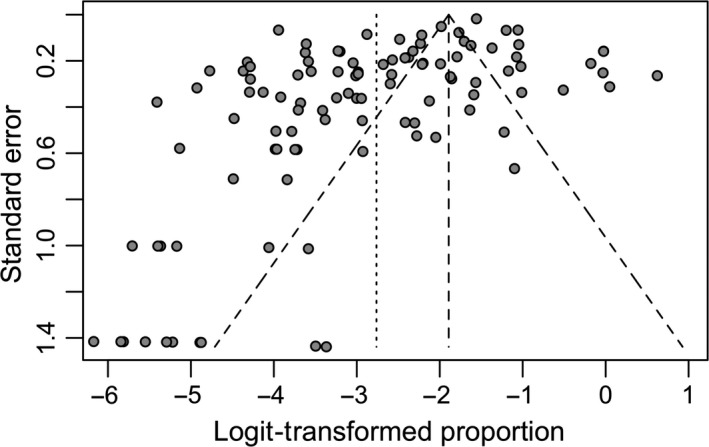
Funnel plot of standard error and logit‐transformed prevalence demonstrates potential publication bias

To assess for potential publication bias, a funnel plot was constructed of the logit prevalence against standard error (Figure 2). The lack of symmetry in the funnel plot illustrates potential publication bias towards smaller studies with lower prevalence. Egger's asymmetry test was significant and showed presence of bias (*p*‐value < 0.001), while Begg's rank correlation test did not (*p*‐value > 0.05).

This evidence of publication bias suggests that RE model will be more appropriate for these data. To explore this further, we estimated both random‐ and fixed‐effects models and constructed funnel plots to compare their fit and look for evidence of systematic bias (Supporting information Figures [Link], [Link]a,b). The RE model demonstrated greater symmetry than the FE model comparatively, suggesting that the RE model is a better fit to the data. Visual inspection of the predicted versus empirical observations (Normal Q‐Q plot) also suggests that the RE model is a better fit to the data (Supporting information Figures [Link], [Link]a,b). As a final check, we constructed receiver operating characteristic (ROC) curves for the two models. The ROC analyses found no difference between the two models in terms of their classification ability (AUC ~ 0.74 for both models).

**Figure 3:**
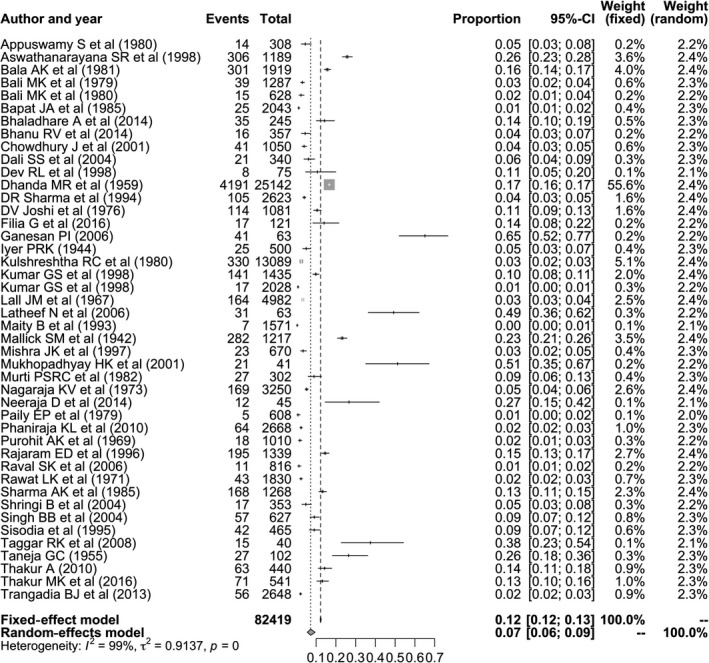
Forest plot visualizing the varying bTB prevalence reported for each included publication in the meta‐analysis. Weightage given to each included publication by both RE and FE models have been shown for rigorous comparison. “Total” refers to the number of animals in each publication, while “Events” refers to the number of bTB‐positive animals. “Proportion” reports the bTB prevalence for each publication

Given the evidence for publication bias and improved qualitative fit of the RE model, we focus on this model, which accounts for heterogeneity between individual studies, to estimate the prevalence of bTB in India from these data. The RE model was estimated from logit‐transformed prevalence rates from individual publications, and the pooled prevalence estimate of bTB in India was determined to be 7.3% (95% CI: 5.6, 9.5). Cochran's (*Q*) value (*Q* = 3939.85, *df *= 105 and *p* < 0.0001) and Higgins statistic (*I* ^*2*^ = 98.9%) were computed to test for heterogeneity. The meta‐analysis, and comparison to the RE model, is graphically summarized in a forest plot (Figure 3).

### 3.3 Meta‐regression

#### 3.3.1 Univariable meta‐regression

Due to the presence of statistical heterogeneity, we conducted univariable meta‐regression in order to determine the effect of study‐level covariates on the estimates of cumulative prevalence. The moderators considered for the analyses were study period, study location, sample size, production system, species, cattle breed and diagnostic technique used.

As seen in Table 3, the proportion of each predictor variable's effect on heterogeneity (*R* ^2^) ranged from 0% to 16.5% in the RE model. Further, under the RE model, the highest value of *R* ^2^ was observed for study location while, diagnostic technique, and sample size exhibited no effect on heterogeneity (*R* ^2^ = 0%).

**Table 3:** Univariable meta‐regression

Predictors	Proportion (*R* ^2^) (%)	*p* value (RE)
Study period	7.0	0.04
Study location	16.5	0.01
Diagnostic technique	0.0	0.70
Species	0.7	0.22
Breed	0.7	0.40
Production system	2.5	0.16
Sample Size	0.0	0.95

*Note*

Proportion of effect of predictors on heterogeneity. All variables had a *p* < 0.01 in the FE model.

#### 3.3.2 Multivariable meta‐regression

All moderators from the univariable meta‐regression were subjected to multivariable meta‐regression (Table 4), which showed that these moderators accounted for 31.4% of the observed heterogeneity. Hence, the significant variables included in our regression model explain only a fraction of the variability observed.

**Table 4:** Multivariable meta‐regression

Predictors	Categories	No. of studies	Odds ratio (95% CI)	*p*‐value (RE)
Study period	1941–1960	7	Reference	
	1961–1980	36	0.15 (0.04, 0.65)	0.01
	1981–2000	29	0.21 (0.05, 1.01)	0.05
	2001–2016	34	0.14 (0.03, 0.65)	0.01
Production systems	Gaushala	6	Reference	
	Organized	71	0.34 (0.09, 1.20)	0.09
	Rural	4	0.24 (0.04, 1.52)	0.13
	Semen station	1	1.05 (0.03, 34.89)	0.98
	Slaughterhouse	9	0.57 (0.06, 5.51)	0.61
Species	Buffalo	23	Reference	
	Cow	83	0.60 (0.28, 1.27)	0.16
Study location	Andhra Pradesh	2	Reference	
	Bihar	1	2.57 (0.13, 52.46)	0.54
	Gujarat	10	0.33 (0.03, 3.59)	0.36
	Haryana	15	0.51 (0.06, 4.48)	0.54
	Himachal Pradesh	3	3.88 (0.30, 49.20)	0.29
	Jammu and Kashmir	1	7.74 (0.27, 218.94)	0.22
	Karnataka	7	1.82 (0.19, 17.33)	0.60
	Kerala	2	0.22 (0.01, 5.80)	0.36
	Madhya Pradesh	5	1.56 (0.14, 17.54)	0.72
	Maharashtra	7	0.81 (0.08, 8.70)	0.86
	Meghalaya	2	1.22 (0.06, 24.31)	0.89
	Orissa	2	0.73 (0.03, 17.07)	0.84
	Pondicherry	1	58.57 (2.16, 1595.91)	0.01
	Punjab	12	2.12 (0.26, 17.49)	0.48
	Rajasthan	6	1.89 (0.18, 19.82)	0.58
	Tamil Nadu	5	8.17 (0.55, 121.89)	0.12
	Uttar Pradesh	16	1.32 (0.15, 11.50)	0.80
	Uttarakhand	2	0.13 (0.01, 3.21)	0.21
	West Bengal	7	2.39 (0.23, 24.87)	0.46
Diagnostic test	SIT	46	Reference	
	Culture	2	3.99 (0.53, 30.28)	0.18
	DIT	25	0.69 (0.23, 2.10)	0.52
	ELISA	2	0.71 (0.09, 5.52)	0.75
	PM Exam	6	0.08 (0.01, 0.77)	0.03
	SICT	11	0.69 (0.18, 2.65)	0.59
	SIE	1	0.07 (0.00, 1.03)	0.05
Breed	Cross‐bred	19	Reference	
	Exotic	10	1.08 (0.37, 3.18)	0.88
	Indigenous	15	0.97 (0.39, 2.37)	0.94
Sample size			1.00	< 0.0001

*Note*

Multivariable meta‐regression of the selected predictors on the prevalence of bTB in India. (*R* ^2^ = 31.4%, *n* = 106).

Analysis of variance (ANOVA) tests indicated that five (study period, study location, species, diagnostic test and breed) of the seven moderators were significant (*p* < 0.25) when the other variables were included (Table 5).

**Table 5:** ANOVA results

Predictors	*p*‐value (RE)
Study period	0.04*
Study location	0.001*
Production system	0.55
Species	0.16*
Diagnostic test	0.12*
Breed	0.13*
Sample size	0.93

*Note*

ANOVA results of individual predictors subjected to multivariable meta‐regression. All variables had a *p* < 0.01 in the FE model. *represents significance.

### 3.4 Effect of moderators on prevalence of bTB

Prevalence estimates using both the RE and FE models are reported in Table 6. As noted above, the values reported from RE model are likely more appropriate given the observed heterogeneity in the studies as the FE model is biased by studies with larger sample size. Based on the RE model, the prevalence of bTB in cows, 6.3% (95% CI: 4.9, 8.0), was marginally higher than the prevalence in buffaloes, 4.3% (95% CI: 2.7, 6.7). Amongst cows, prevalence by breed did not vary greatly as cross‐bred cows were found to have the highest prevalence with 8.1% (95% CI: 4.6, 13.8), followed by indigenous cows with 7.4% (95% CI: 4.0, 13.1), and exotic cows with 7.0% (95% CI: 3.7, 12.9). Unlike cattle breed, larger differences were seen amongst production systems as cattle housed in Gaushalas (protective shelters for unproductive or destitute cows in India) had a higher prevalence, 19.1% (95% CI: 13.0, 27.1) than those kept in organized farms, 5.1% (95% CI: 3.8, 6.7) and rural conditions, 4.4% (95% CI: 1.0, 16.5). The time period, 1941–1960, was found to have the highest prevalence, 13.8% (95% CI: 10.5, 17.9), while 1961–1980 was found to have the lowest, 3.6% (95% CI: 2.6, 4.9). A total of 28,073 animals had been tested during 1961–1980. The time period between 1981 and 2000 showed a prevalence of 7.0% (95% CI: 4.8, 10.2), and the prevalence of the most recent time period between 2001 and 2016 was determined to be 6.8% (95% CI: 4.3, 10.7) (Table 6).

**Table 6:** Pooled prevalence estimates (derived from both RE and FE models) of the various predictors namely, cattle species, breed, production system and study period

	Predictors	Sample size	Prevalence (95% CI) (RE model)	Prevalence (95% CI) (FE model)
Species	Buffalo	29,037	4.3% (2.7, 6.7)	16.0% (15.5, 16.4)
	Cow	53,382	6.3% (4.9, 8.0)	10.2% (9.8, 10.5)
Cattle breed	Exotic	2,011	7.0% (3.7, 12.9)	16% (14.1, 18.2)
	Cross‐bred	9,548	8.1% (4.6, 13.8)	13.5% (12.7, 14.5)
	Indigenous	4,169	7.4% (4.0, 13.1)	15.5% (14.0, 17.1)
Production systems	Gaushala	576	19.1% (13.0, 27.1)	18.7% (15.7, 22.3)
	Organized farm	43,847	5.1% (3.8, 6.7)	8.4% (8.1, 8.7)
	Rural farm	1,607	4.4% (1.0, 16.5)	3.3% (2.2, 4.7)
Study period	1941–1960	26,961	13.8% (10.5, 17.9)	17.0% (16.6, 17.5)
	1961–1980	28,073	3.6% (2.6, 4.9)	3.9% (3.6, 4.2)
	1981–2000	16,927	7.0% (4.8, 10.2)	13.9% (13.2, 14.6)
	2001–2016	10,458	6.8% (4.3, 10.7)	9.2% (8.5, 10.0)

### 3.5 Geographical distribution of included studies in India

**Figure 4:**
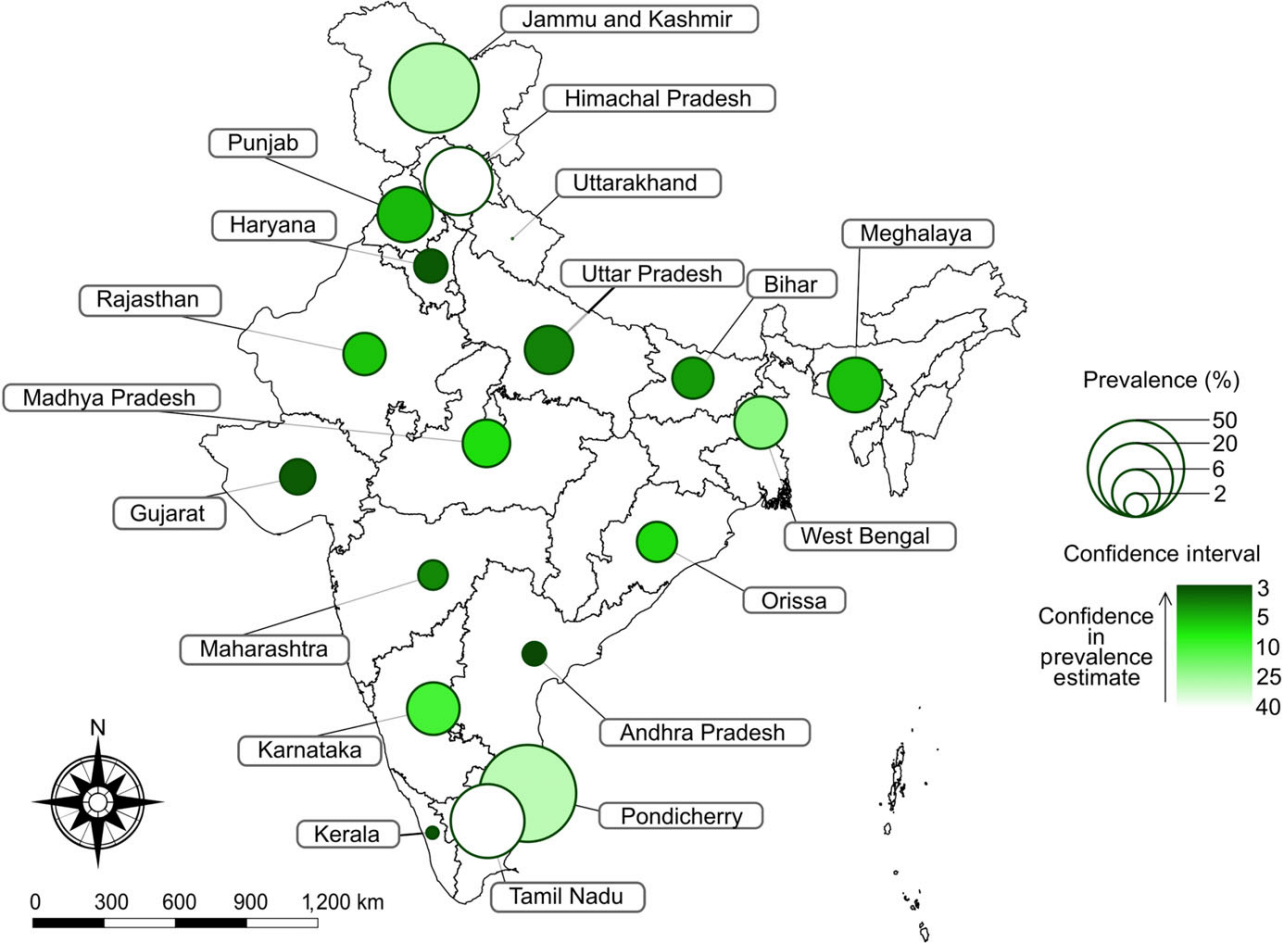
Geographical distribution and pooled prevalence estimates (RE model) of bTB in the different states of India. For the confidence intervals, a power scale was used to map colour lightness and represented as *y* = mx^*k*^ + *b*, where *k* = 0.5. Prevalence was mapped to a log‐scale where data were uniformly corrected with allow for visual properties. Note that although scales were altered, the original data set is provided in Table 7 to afford accurate measures [Colour figure can be viewed at http://wileyonlinelibrary.com]

Study reports from included publications encompassed 18 states and one union territory in India. No reports were found for Arunachal Pradesh, Assam, Chhattisgarh, Goa, Jharkhand, Manipur, Mizoram, Nagaland, Sikkim, Telangana, Tripura, Andaman and Nicobar Islands, Chandigarh, Dadra and Nagar Haveli, Daman and Diu, Delhi, and Lakshadweep, comprising a total of 11 states and six union territories. It can be observed from the map that the prevalence of bTB varied highly between states (Figure 4) (Table 7).

**Table 7:** Pooled prevalence estimates (RE model) of bTB prevalence in India by state

STATE	Sample size	Prevalence (95% CI) (RE model)
Andhra Pradesh	426	2% (1.0, 3.9)
Bihar	169	4.7% (2.4, 9.2)
Gujarat	28,268	3.6% (2.2, 5.8)
Haryana	17,693	3.3% (1.9, 5.4)
Himachal Pradesh	617	15.4% (4.2, 43.4)
Jammu and Kashmir	40	37.5% (24.0, 53.2)
Karnataka	7,152	7.9% (3.0, 19.2)
Kerala	608	1.0% (0.3, 3.6)
Madhya Pradesh	2,295	6.3% (2.7, 14.00)
Maharashtra	2,697	2.7% (1.0, 6.9)
Meghalaya	302	8.7% (5.1, 14.3)
Orissa	670	4.5% (1.5, 12.5)
Pondicherry	41	51.2% (36.3, 66.0)
Punjab	5,780	8.9% (5.5, 14.2)
Rajasthan	2,165	5.0% (2.1, 11.5)
Tamil Nadu	1,822	19.6% (6.6, 45.9)
Uttar Pradesh	8,156	6.5% (4.3, 9.8)
Uttarakhand	227	0.4% (0.1, 3.1)
West Bengal	3,291	7.8% (2.1, 25.7)
Grand Total	82,419	

## 4 DISCUSSION

After screening of 285 publications, we extracted data from 44 cross‐sectional studies published in peer‐reviewed journals that report the prevalence of bTB in India and conducted meta‐analysis. The pooled prevalence estimate (RE model) for all of India was found to be 7.3% (95% CI: 5.6, 9.5). Despite being a disease of antiquity with significant animal and public health costs that have been controlled in most developed countries over a half‐century ago, bTB has a high and widespread prevalence in India as no national control strategies have been implemented in the country (Figure 4). These data suggest that India, as the world's largest producer of milk (~156 MMT), accounting for ~18.5% of the world's total milk production and the world's largest red meat exporter (~1.9 MMT), has an urgent and as yet unmet need for control of bTB for both economic and public health reasons (DADF, 2015).

Following the White Revolution (Bellur, Singh, Chaganti, & Chaganti, 1990) (Nair, 1985), a rural development programme in India that resulted in making India the largest producer of milk and milk products, organized dairy farming has expanded rapidly. These farms have comparatively high (and still increasing) animal densities, paving the way for higher probabilities of disease transmission. In contrast, rural farms are owned by small‐holder farmers and have much lower stocking densities, resulting in lower likelihoods of disease transmission. Our results show the prevalence of bTB in animals housed under organized farming systems to be 5.1% (95% CI: 3.8, 6.7) and rural conditions to be 4.4% (95% CI: 1.0, 16.5). The overlap of CI in prevalence between organized and rural settings as suggested by the RE model is curious given the prevailing dogma that organized farming poses higher risk of disease transmission, suggesting that further investigation is needed for clarification of this issue. Yet, amongst the three different production systems in which the animals were housed, Gaushalas had the highest prevalence of bTB, 19.1% (95% CI: 13.0, 27.1). Gaushalas are protective shelters for destitute or unproductive cows in India. There are over 5,100 of these “old age homes” for cows in India (DADF, G. o. I., 2015), which may account for the higher prevalence observed in this group of animals as bTB is a chronic infection. While noteworthy and in line with expectations of observing greater prevalence in such a high‐risk setting, the lower sample size for Gaushalas compared with sample sizes of other production systems must be kept in consideration and warrants further investigation.

Overall, the ordering of prevalence estimates determined using the FE model for different production systems follows the same trend as in the RE model (i.e., prevalence in Gaushala > Organized farms > Rural farms) (Table 6). However, given the observed heterogeneity in the studies, it is difficult to assess the validity of the FE model, and hence, further study is necessary to clarify the exact influence that each production system has on bTB prevalence before definitive conclusions can be made. We note that accurate estimates of prevalence rates for each production system are particularly important in the Indian context where the magnitude of animals housed in Gaushalas and the increasing population of cattle being reared under intensive conditions have the potential to considerably impact overall prevalence and influence assessment of bTB transmission rates and targeted interventions.

Regarding animal species (cow versus buffalo), the meta‐analysis (RE model) shows prevalence to be higher in cows [6.3% (95% CI: 4.9, 8.0)] than in buffaloes [4.3% (95% CI: 2.7, 6.7)]. However, we note that the prevalence in buffaloes determined using the FE model was 16.0% (95% CI: 15.5, 16.4) and that in cows was 10.2% (95% CI: 9.8, 10.5). The high prevalence observed in buffaloes using the FE model is most likely driven by a single study that sampled 21,592 buffaloes (of a total buffalo sample size of 29,037 included in this meta‐analysis) and recorded a prevalence of 17.4% (Dhanda & Lall, 1959). As per the Government of India's Department of Animal, Dairy and Fisheries (DADF) 2016–2017 Annual report, the share of milk contribution from buffaloes is 49% and that of cows is 48% (DADF, 2016). Assuming a conservative 10% loss in milk productivity due to bTB (Thoen, 2008) and the overall estimated bTB prevalence rates based on the RE model, the annual costs to farmers only from loss in milk production in cows and buffaloes in India are estimated to range from 375 to 544 million USD (Supporting information Table S1). We note that the need for intensification of dairy production to meet increased milk demand and national priorities for nutritional improvement and rural development is likely to significantly increase bTB disease prevalence as the disease is known to more easily spread amongst intensively reared cattle. With the inevitable increase in bTB prevalence, this already large economic cost will only continue to grow if no intervention measures are implemented.

Published studies on the influence of breed on genetic susceptibility to bTB showed that the native breed of cattle is more resistant to the disease than exotic breed (Vordermeier et al., 2012) (Soparkar, 1925) (Liston & Soparkar, 1917) (Sharma, Vanamayya, & Parihar, 1985), affirming a generally held and commonly disseminated dogma. In contrast, our results note no significant differences in bTB prevalence between cow breeds in either the RE or the FE models (Table 3). However, given the heterogeneity observed in the studies, rigorous investigations of the true differences in susceptibility amongst different cattle breeds to bTB will be essential for evidence‐based formulation of a rational approach to control this disease in India.

India has a cattle population of nearly 300 million, and we attempted a conservative estimation of the number of infected cattle in India. As per DADF, G. o. I., 2015 (Basic Animal Husbandry and Fisheries Statistics, 2015), there were 39.7 m exotic and cross‐bred cows, 151.2 m indigenous cows and 108.7 m buffaloes in 2015 (DADF, G. o. I., 2015). Applying bTB prevalence estimates (obtained from our meta‐analysis) of 7.3% (95% CI: 5.6, 9.5) across all cattle types, there are likely to be ~21.8 m (95% CI: 16.6, 28.4) bTB‐positive cattle in India, suggesting that India likely also has the highest burden of bTB‐infected animals in the world, exceeding even at the lower confidence interval the total number of dairy cattle in the United States (USDA, 2016). We note that *M. bovis* may not necessarily be the only causative agent of bTB in all reactor animals as isolation of *M. tuberculosis* from cattle samples has also been reported (Srivastava et al., 2008) (Sweetline Anne, Ronald, Kumar, Kannan, & Thangavelu, 2017).

The multivariable logistic regression model accounted for ~31% of the heterogeneity between studies, suggesting that additional factors not part of our model are also contributors to bTB prevalence. These factors may include animal age, sex and herd size that were not represented with enough frequency in the papers included in the systematic review to be subject to robust and rigorous meta‐analysis. Hence, future studies should strive to understand how these factors contribute to overall bTB prevalence.

We note that the findings of this study must be considered in conjunction with the limitations inherent in systematic reviews and meta‐analyses. For example, studies were limited to those included in the four databases used and no single study sample can provide a perfect representation of the cattle in a state or across the country. Further, our review consisted of only published studies written in English and did not capture any unpublished data, subjecting our analysis to publication bias (Figure 4). This potential publication bias is supported by the significant result of Egger's regression test, the test with greater statistical power (Hayashino, Noguchi, & Fukui, 2005).

In addition, our findings are limited by the variation in experimental design and methodology of each included article. Variation in the reporting details of each article also contributes to variations in study quality. It is important to note that the pooled prevalence estimate of 7.3% (95% CI: 5.6, 9.5) was derived from studies that, despite efforts during the screening process to exclude studies with biased sampling methods, may not have been entirely random surveys and needs to be refined with proper cross‐sectional national level surveys using internationally recognized and well‐standardized methods. In addition, acceptance of each study's reported number of positive animals as truly positive is also a limiting but unavoidable reality of lacking access to the original data and an inability to know the sensitivity, specificity and other performance characteristics of the tests used. In addition, while most studies’ objectives were to determine bTB prevalence in the study population, studies differed in their examination or reporting of specific moderators of interest.

Our analysis also indicated the presence of temporal heterogeneity (*R* ^2^ = 7.04%) over the 74‐year time frame (1942–2016) represented by the included studies (Table 3). While the specific source(s) of this heterogeneity is unclear, contributors may include differences in environmental conditions over time (Humblet et al., 2010) (Bekara, Azizi, Bénet, & Durand, 2016), the number of studies within each time interval, animals tested, test operators’ skills/methods and the diagnostic tests themselves. Recent studies have also shown that the quality, origin and source of tuberculin used are variable within tuberculin‐based tests, highlighting a lack of standardization (Bakker et al., 2005). In addition to such variation within individual tests, the performance, sensitivity and specificity vary across tuberculin‐based tests making comparisons difficult and imprecise (Hartnack & Torgerson, 2012) (Varello et al., 2008) (Cousins & Florisson, 2005) (Ameni, Miörner, Roger, & Tibbo, 2000). While most tests are tuberculin‐based, there are potential causes for heterogeneity that remain to be explored. Thus, combined with the existing limitations of non‐standardized and varying performance characteristics of current diagnostic tests, we underscore the need for a national surveillance programme using a single, well‐standardized skin test performed by independent, well trained operators using OIE approved protocols and well‐standardized tuberculin antigen to enable accurate monitoring of bTB prevalence over time and the impacts of any potential intervention or control programme.

Several previous studies have reported prevalence of bTB in farms that used control strategies for the disease. Although test and slaughter of reactor animals as a control strategy are practically impossible in developing countries like India due to both economic and social considerations, the above‐mentioned studies provide preliminary evidence that even test, and segregate approaches have the potential to help reduce the prevalence of bTB in India, at least in intensively farmed animals that are regularly tested using well‐standardized tests (Dhanda & Lall, 1959) (Krishnaswamy, Nagaraja, Keshavamurthy, Nanjiah, & Adinarayanaiah, 1973; Mukherjee, 2006).

Taken together, the meta‐analysis highlights a critical and hitherto unmet need for the development of a national surveillance programme and the implementation of an effective strategy for control of bTB in India—a need that will only continue to grow in conjunction with India's increasing cattle population and demands on milk production and an inability to cull potentially diseased cows. Given the likely inability of implementing a test and cull programme at any scale due to social and economic considerations, the need for a vaccine that can reduce the burden of infection and transmission is critical. In this context, we note that recent reports suggest that the century‐old BCG vaccine may have considerable utility in this regard (Ameni, Vordermeier, Aseffa, Young, & Hewinson, 2010) (Ameni et al., 2017), but requires further study to evaluate its ability in reducing onward transmission. However, if effective, there is also an unmet need for a validated and accepted fit‐for‐purpose DIVA (Differentiating Infected from Vaccinated Animals) diagnostic test for the detection of bTB‐infected cattle that can be used in conjunction with a vaccine programme.

*Mycobacterium bovis* has also been isolated from milk samples of tuberculous cattle (Aswathanarayana, Rao, Krishnappa, Ramanatha, & Raghavan, 1998) (Veerasami et al., 2012). Given the fact that over 70% of the milk in India is sold unpasteurized (FAO/OIE/WHO, 1993), this raises concerns regarding the potential for zoonotic transmission of bTB and continued spread of human tuberculosis (India has the world's largest burden of human TB) (Thoen, LoBue, & de Kantor, 2006). In May 2014, the World Health Assembly adopted a new strategy to attain an ambitious goal of ending the global TB epidemic by 2035: the End TB strategy (Uplekar et al., 2015). Given the prevalence of bTB and the potential for zoonotic transmission, particularly to children and others who consume unpasteurized or unprocessed milk from infected cows, there is a critical need for a national bTB control programme in India and other developing countries as attempts to eradicate the disease from humans without eradicating it from cattle are likely to prove futile. Importantly, implementation of a national control programme would not only enable accurate temporal trends and estimates of bTB prevalence, risk and economic costs, but would equally importantly improve the health and productivity of cattle in India.

## 5 CONCLUSION

Overall, the results of our systematic review and meta‐analysis conducted on 44 publications indicate high and widespread bTB prevalence in India of 7.3% (95% CI: 5.6, 9.5). Further study is necessary to obtain more robust state‐by‐state prevalence estimates and explore other moderators of risk (including herd size, animal sex, and age, amongst others) that are likely to impact development and implementation of a rational and effective bTB control strategy. Taken together with the expected dairy farm intensification, growing demands for increased milk production and the zoonotic nature of *M. bovis*, the results of our current studies highlight the importance of developing and implementing a national bTB control programme that will need to include a national surveillance plan using (a) well‐standardized method(s) and evidence‐based intervention(s) that are likely to work in India and other developing country settings.

## CONFLICT OF INTEREST

The authors have no conflict of interest.
