# Supplementary figures and images for: Prevalence of Bovine Tuberculosis in India: A systematic review and meta‐analysis
Source: Transbound Emerg Dis. 2018 Jun 8;65(6):1627–40. doi: 10.1111/tbed.12915 (PMC6282864; doi:10.1111/tbed.12915)

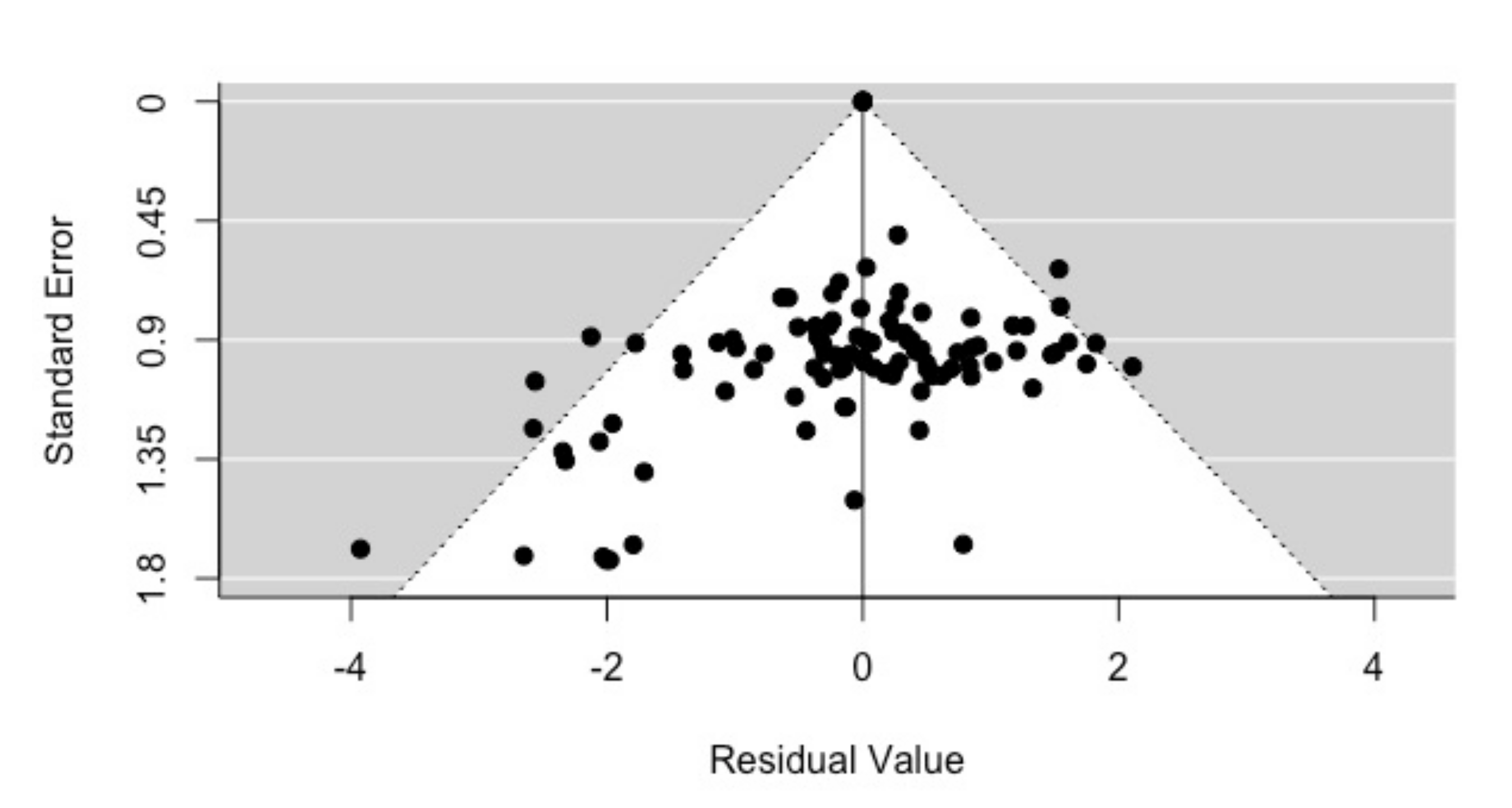

Supplement: Supplementary file 1 [file TBED-65-1627-s001.tiff]

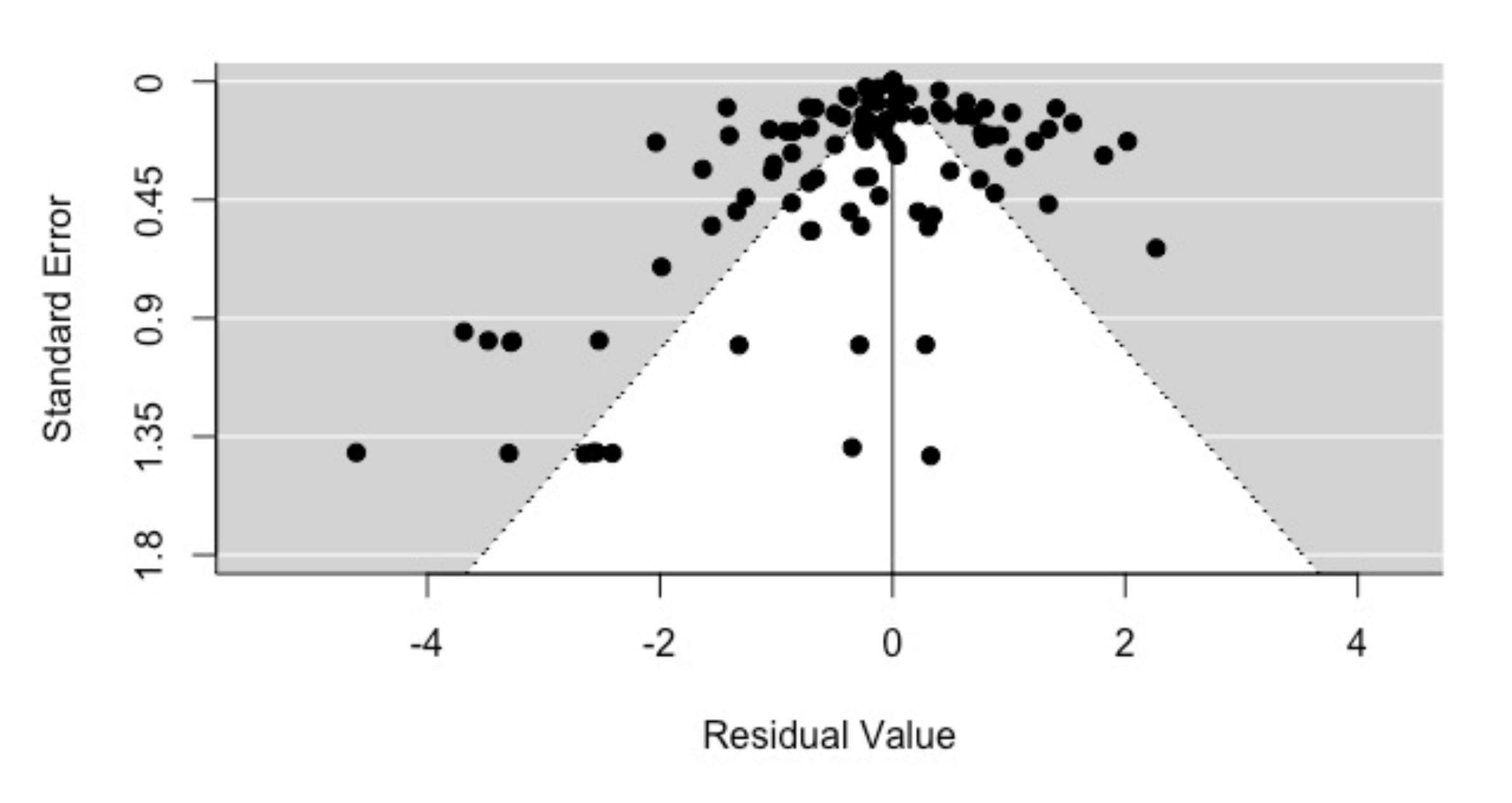

Supplement: Supplementary file 2 [file TBED-65-1627-s002.tiff]

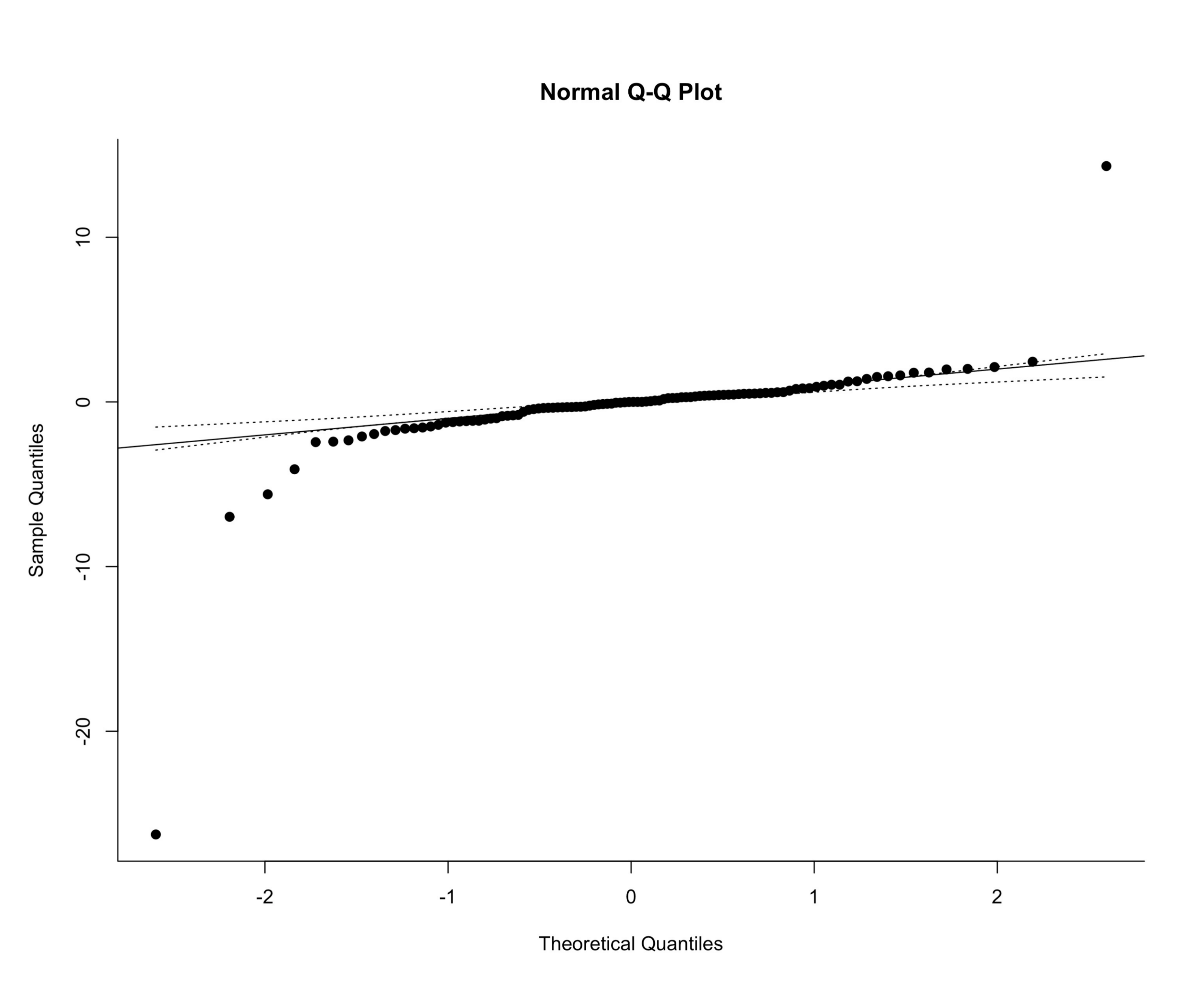

Supplement: Supplementary file 3 [file TBED-65-1627-s003.tiff]

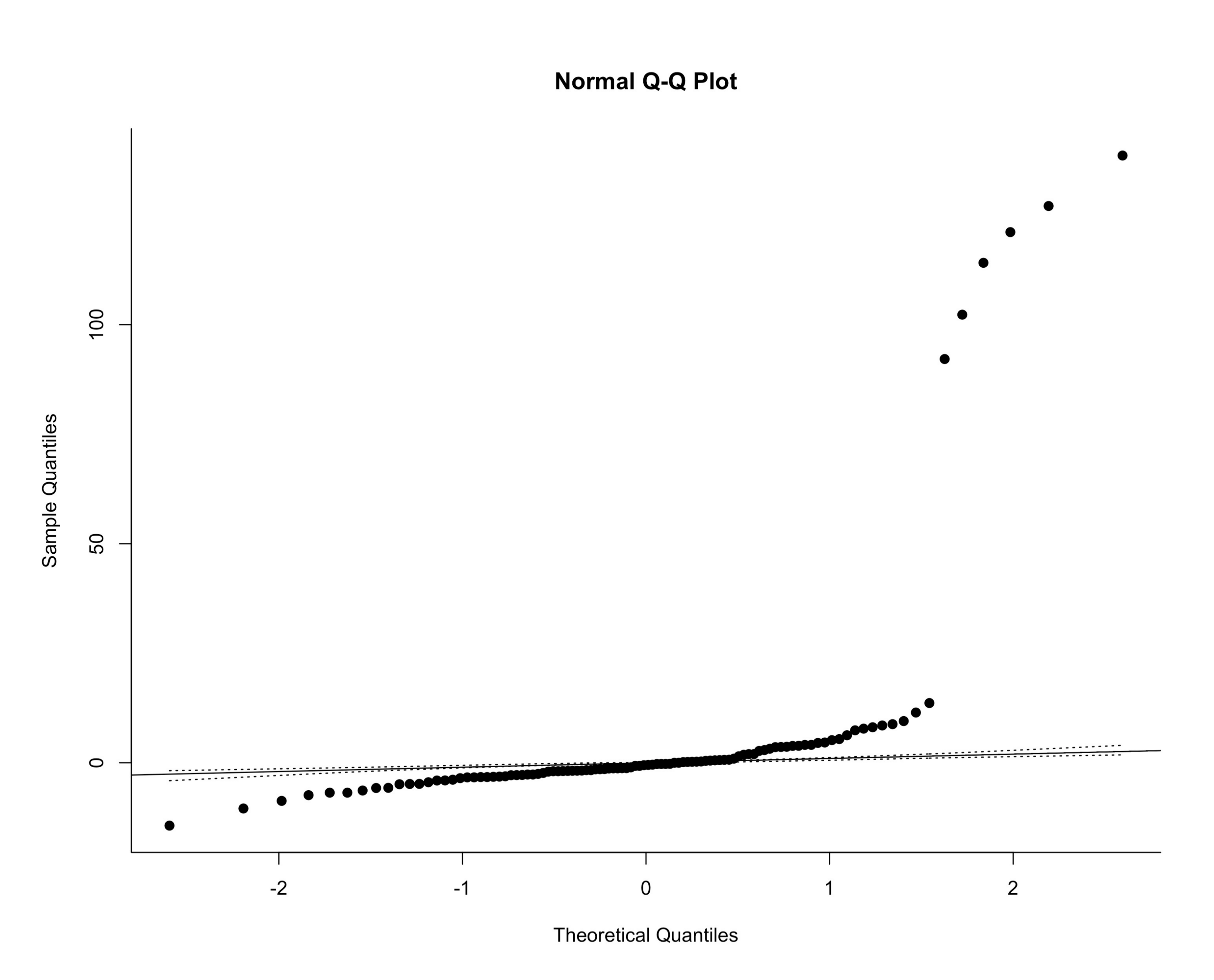

Supplement: Supplementary file 4 [file TBED-65-1627-s004.tiff]
