# Supplementary material for: Prevalence of Bovine Tuberculosis in India: A systematic review and meta‐analysis
Source: Transbound Emerg Dis. 2018 Jun 8;65(6):1627–40. doi: 10.1111/tbed.12915 (PMC6282864; doi:10.1111/tbed.12915)
Supplement: Supplementary file 5 [file TBED-65-1627-s005.docx]

| **Supplementary Table 1.** Current (2017) cost of milk loss due to bTB | | | | | | |
| --- | --- | --- | --- | --- | --- | --- |
| **Cattle Type** | **Estimated Prevalence (%)** | **% Milk share (2017)** | **Volume contribution (MT)** | **Value (USD. '000 million)** | **Loss (USD. million) assuming indivual species prevalence** | **Loss (USD. Million) assuming pooled 7.27% bTB Prevalence (RE Model)** |
| **Buffalo (Non-descript and Indigenous)** | 4.3% | 49 | 76 | 47.09 | 201.55 | 342.34 |
| **Cow (Cross-bred, Indigneous and exotic)** | 6.3% | 48 | 75 | 27.8 | 174.03 | 202.11 |
| **Totals** |  |  | 151 | 74.89 | 375.57 | 544.45 |
| Current (2017) loss is calculated as (Value of milk produced) x (Estimated prevalence of bTB) x (Estimated reduction in milk production due to infection); Assumptions based on literature include – avg. cow milk price = USD 0.27 /kg; avg. buffalo milk price = USD 0.62 /kg; and reduction in milk production due to bTB infection is conservatively estimated to be 10%. | | | | | | |
